# Revealing large metagenomic regions through long DNA fragment hybridization capture

**DOI:** 10.1186/s40168-017-0251-0

**Published:** 2017-03-14

**Authors:** Cyrielle Gasc, Pierre Peyret

**Affiliations:** Université Clermont Auvergne, INRA, MEDIS, 63000 Clermont-Ferrand, France

**Keywords:** Large genomic regions reconstruction, Metagenomics, Sequence capture by hybridization, Bioremediation

## Abstract

**Background:**

High-throughput DNA sequencing technologies have revolutionized genomic analysis, including the de novo assembly of whole genomes from single organisms or metagenomic samples. However, due to the limited capacity of short-read sequence data to assemble complex or low coverage regions, genomes are typically fragmented, leading to draft genomes with numerous underexplored large genomic regions. Revealing these missing sequences is a major goal to resolve concerns in numerous biological studies.

**Methods:**

To overcome these limitations, we developed an innovative target enrichment method for the reconstruction of large unknown genomic regions. Based on a hybridization capture strategy, this approach enables the enrichment of large genomic regions allowing the reconstruction of tens of kilobase pairs flanking a short, targeted DNA sequence.

**Results:**

Applied to a metagenomic soil sample targeting the *linA* gene, the biomarker of hexachlorocyclohexane (HCH) degradation, our method permitted the enrichment of the gene and its flanking regions leading to the reconstruction of several contigs and complete plasmids exceeding tens of kilobase pairs surrounding *linA*. Thus, through gene association and genome reconstruction, we identified microbial species involved in HCH degradation which constitute targets to improve biostimulation treatments.

**Conclusions:**

This new hybridization capture strategy makes surveying and deconvoluting complex genomic regions possible through large genomic regions enrichment and allows the efficient exploration of metagenomic diversity. Indeed, this approach enables to assign identity and function to microorganisms in natural environments, one of the ultimate goals of microbial ecology.

**Electronic supplementary material:**

The online version of this article (doi:10.1186/s40168-017-0251-0) contains supplementary material, which is available to authorized users.

## Background

Over the past few years, the emergence of high-throughput sequencing methods has revolutionized the exploration of biological samples; these methods have made genomic sequence information easily accessible. With continually decreasing costs and constant technical improvements, such as the length and number of reads generated, these methods are increasingly used for genomic studies [1]. Dedicated bioinformatics tools have been developed for the analysis of the large numbers of short reads obtained. Nevertheless, despite methodological and computational improvements, data treatment remains a particularly challenging task [2–4]. The reconstruction of complete genomes from next-generation sequencing (NGS) data, even for single organisms, is difficult because of the short read lengths, the uneven sequencing depths, and the repeat patterns; most of the genomes currently published are permanent drafts. Thus, the quality of most genomes is well below the sequence contiguity and accuracy achieved by clone-ordered reference genomes, albeit at far less cost [2]. Few modern genome assemblies exceed an N50 contig of 100 kbp (average of 41 kbp). This translates into tens to hundreds of thousands of sequence gaps. It is currently estimated that 5–40 Mb of euchromatic sequences are absent from a given human reference genome owing to structural polymorphism and that an additional 125–150 Mb of gene-rich regions of the genome are inaccessible to standard variation analyses. Access to underexplored but relevant fractions of genomes, regardless of the organism, is a major goal to resolve important concerns in medical, biotechnical, evolutionary, and ecological studies. In ecological studies, metagenomic sample exploration is particularly difficult. Microbial communities possess the greatest organism diversity on earth; species show different degrees of phylogenetic proximity and highly variable abundances [5, 6]. For example, 1 g of soil is estimated to contain up to 10^9^ bacterial cells belonging to 10^6^ distinct prokaryotic taxa [7, 8]. Despite the development of devoted tools, the reconstruction of microbial genomes from these samples is only sustainable for the most abundant microorganisms [9, 10]. The comprehension of microbial community functioning is consequently reduced because of the inability to associate with certainty through genome assembly, the identities of the microorganisms present and the specific metabolic functions they perform.

To overcome these limitations related to genome reconstruction and to facilitate genomic diversity exploration of model and non-model organisms, we developed a target enrichment method for large genomic regions based on hybridization capture that could be applied to single organisms or metagenomic samples. Gene capture approaches by hybridization traditionally use tiling probes for resequencing experiments to identify new genetic variants [11]. Based on the potency of sequence capture for expanding knowledge beyond target DNA regions, studies have used hybridization capture strategies to study the unknown flanking regions of genes or particular genomic regions [1]. However, flanking DNA regions recovered are of small size (approximately 200 bp) due to the size of NGS libraries used. Indeed, such libraries consist of short fragments of DNA specifically selected for optimal performance with NGS platforms. With such a method applied on metagenomic samples, we captured flanking regions that do not exceed 1 kbp [12]. Other work demonstrated the possibility to enrich several kilobase pairs of flanking regions from simple models with hybridization capture, but this approach has proved to be inefficient when applied to complex genomes [13]. Another study combined third-generation sequencing and hybridization capture to enrich DNA fragments of several kilobase pairs from simple models [14]. Although, the use of tiling probes allowed to discover new sequence variants in region covered by probes that needed to be confirmed by other methods, this strategy could not reveal unknown flanking sequences. Another strategy used short primers that are enzymatically extended incorporating biotinylated nucleotides allowing the capture of produced DNA fragments [15]. However, this strategy is more adapted to enrich DNA fragments of few kilobase pairs because of efficiency which drops drastically with little to no difference between targeted and non-targeted DNA fragments of only 20 kbp long.

To enrich large genomic regions flanking a targeted biomarker, we developed an innovative sequence capture method relying on the construction of high-quality, size-selected large-insert capture libraries. This approach thus enables the direct enrichment of fragments of tens of kilobase pairs with the use of short non-overlapping probes targeting a few base pairs of DNA sequence. Thereby, for the first time, with this new method, we demonstrate that it is possible to easily, specifically, and efficiently reconstruct unknown flanking genomic regions of tens of kilobase pairs flanking a short, targeted DNA sequence. Due to the large DNA fragments captured, this innovative approach allows the resolution of complex genetic organization with the presence of repeats or genetic variations that are highly difficult to characterize using current molecular approaches. When applied to metagenomic samples, this approach enables the assignment of identity and function to microorganisms in natural environments, one of the ultimate goals of microbial ecology.

## Methods

### DNA isolation

Genomic DNA samples were extracted from *Sphingobium indicum* B90A (DSMZ No 16412) and *Roseobacter denitrificans* OCh 114 (DSMZ No 7001) liquid cultures using a Blood & Cell Culture DNA kit (Qiagen) and were mixed in equimolar concentrations.

A hexachlorocyclohexane (HCH)-contaminated soil sample was collected from an old chemical factory (Huningue, France), and genomic DNA extraction was performed using a PowerSoil DNA Isolation Kit (MoBio).

### Probe design and synthesis

For probe design, 145 *linA* nucleic sequences, corresponding to the *linA1*, *linA2*, and *linA3* types [16], have been extracted from GenBank (December 18, 2014). A set of four 80-mer degenerate non-overlapping probes was designed from these sequences using KASpOD software [17] that allows for the design of specific probes based on large sequence datasets (Additional file 1: Table S1). The four selected probes are distributed uniformly over the entire length of the *linA* gene and can hybridize all the known *linA* sequences used for the probe design and potentially other *linA* variants never described in databases. Probe specificity has been verified using the BLAST program against an exhaustive database containing approximately 10 million microbial coding data sequences (CDS) from the EMBL databank.

Adaptor sequences were added at each extremity of the probe to enable their PCR amplification, resulting in “ATCGCACCAGCGTGT-N_80_-CACTGCGGCTCCTCA” sequences, with N_80_ representing the *linA*-specific capture probes. Biotinylated RNA capture probes were then synthesized as described by Ribière et al. [18]. In brief, adaptors containing the T7 promoter were added to probes through ligation-mediated PCR, and final biotinylated RNA probes were obtained after in vitro transcription and purification.

### Targeted capture

For long DNA fragment library construction, 4 μg of genomic DNA extracted from bacterial strains and from soil was sheared to 6- and 20-kbp fragments, respectively, using Covaris g-TUBES. Fragments were size selected through 0.75% agarose pulsed field gel electrophoresis migration with BluePippin (Sage Science) using 5,000–7,000 and 18,000–22,000 bp size ranges, respectively. The size-selected fragments were then amplified during 2 h at 30 °C and 10 min at 65 °C with the IllustraGenomPhi V2 DNA Amplification kit (GE Healthcare). To mitigate biases, we limited the multiple displacement amplification (MDA) time as previously described by Sangwan et al. [19] and applied the MDA on the size-selected DNA fragments rather than whole metagenome to reduce chimera formation. Amplified DNA fragments were size selected again with BluePippin using the parameters previously described. Library quality and size distribution were evaluated on an Agilent Bioanalyzer chip and concentration was estimated using Qubit dsDNA HS kit (Invitrogen).

For each capture experiment, 2.5 μg of salmon sperm DNA (Ambion) and 500 ng or 2 μg of denatured DNA from bacterial strains and from soil, respectively, were mixed, denatured for 5 min at 95 °C, and incubated for 5 min at 65 °C before adding 13 μl of prewarmed (65 °C) hybridization buffer (10× SSPE, 10× Denhardt’s Solution, 10 mM EDTA and 0.2% SDS) and 500 ng of prewarmed (65 °C) biotinylated RNA probes. After hybridization at 65 °C for 24 h, the probe/target heteroduplexes were captured using 500 ng of washed streptavidin-coated paramagnetic beads (Dynabeads M-280 Streptavidin, Invitrogen). The beads were precipitated with a magnetic stand (Ambion) and washed once at room temperature with 500 μl 1× SSC/0.1% SDS and three times at 65 °C with 500 μl prewarmed 0.1× SSC/ 0.1% SDS. The captured fragments were eluted with 50 μl of 0.1 M NaOH. After magnetic bead precipitation, the DNA supernatant was transferred to a sterile tube containing 70 μl of 1 M Tris–HCl pH 7.5. The long DNA fragments captured were then purified through centrifugation (20 min at 500*g* followed by 3 min at 1000*g*) using Microcon DNA Fast Flow Centrifugal Filters (Merck Millipore). Purified fragments were amplified with the IllustraGenomPhi V2 DNA Amplification kit and size selected with BluePippin as described before. Quality and size distribution of long DNA fragments captured were evaluated on an Agilent Bioanalyzer chip, and concentration was estimated using Qubit dsDNA HS kit (Invitrogen).

### Sequencing

DNA directly extracted from soil and captured DNA products from bacterial strains and soil were sequenced in two MiSeq 2 × 300 bp runs (Illumina) after Nextera library construction according to the manufacturer’s instructions. Thus, during this step, large genomic fragments obtained after hybridization capture were cut to short DNA fragments (approximately 400 bp).

Data files are publicly available through the NCBI Sequence Read Archive (SRA; http://www.ncbi.nlm.nih.gov/sra) under accession numbers SRR3545174 (hybridization capture on the artificial mixture of strains), SRR3546812 (hybridization capture on the soil sample), and SRR3546814 (shotgun sequencing on the soil sample).

### Data processing

All raw reads were scanned for library adaptors and quality filtered using the PRINSEQ-lite PERL script [20] prior to analysis. After pretreatment, 664,854 pairs of reads were obtained for the capture of the bacterial strains mixture, 15,141,059 were obtained for the soil sample capture, and 19,377,512 were obtained for the soil shotgun sequencing.

Reads obtained from hybridization capture and the direct sequencing of soil DNA were mapped against reference genomes using Bowtie2 (V2.1.0) [21] with end-to-end sensitive mode, and no mismatch was allowed in 28-bp seed alignments. Coverage at each genome position and the number of reads aligned in determined regions were calculated using SAMtools 1.3 [22], and genome coverage with reads mapped was represented using Circos 0.69 [23].

Quality-controlled reads obtained from hybridization capture on the soil sample were de novo assembled using IDBA-UD (v1.1.2) [24] with kmers ranging from 20 to 100 and an increment of 20 at each iterative process. Contigs were then subjected to a second round of assembly using CAP3 [25] with default parameters to obtain longer contigs. The longest (≥1,000 bp) and most covered (≥5000 reads) contigs enriched through hybridization capture were aligned with blastn against the NCBI representative genomes (May 28, 2014) and wgs (October 6, 2014) databases.

## Results

We applied our hybridization capture approach to target and reconstruct the flanking regions of the 471-bp *linA* gene, which encodes a dehydrochlorinase involved in the first steps of the degradation of hexachlorocyclohexane (HCH), a chemical pesticide that was widely used for years to control agricultural pests and now prohibited in many countries because of its toxicity and persistence in the environment [26, 27]. On the basis of all *linA* nucleic sequences available in databases, we designed a set of four non-overlapping degenerated 80-mer probes capable of targeting all known genes belonging to *linA1*, *linA2*, and *linA3* types [16] and potentially unknown *linA* variants (Additional file 1: Table S1). We then synthesized the single-strand RNA biotinylated probes in high quantities through in vitro transcription and used them for hybridization capture.

### Validation of hybridization capture on a simple biological model

We first validated our approach on an artificial mixture of two bacterial strains to evaluate its efficiency with simple models and its capacity to reconstruct large regions even with highly fragmented sequence information. We targeted the *linA* gene in an equimolar mixture of DNA of two bacterial strains: *Sphingobium indicum*, a model species for HCH degradation carrying one copy of the targeted gene on its genome; and *Roseobacter denitrificans*, a phylogenetically distant species with no *linA* gene or any HCH degradation capability. We used our set of biotinylated probes to hybridize long DNA fragments containing *linA* coding sequence from a bacterial DNA mixture that had been sheared to 6-kbp fragments (Additional file 1: Figure S1). We recovered the probe-long fragment heteroduplexes with streptavidin-coated magnetic beads, and after elution, linear amplification, and paired-end library construction, we sequenced the captured DNA fragments on an Illumina MiSeq platform.

**Fig. 1:**
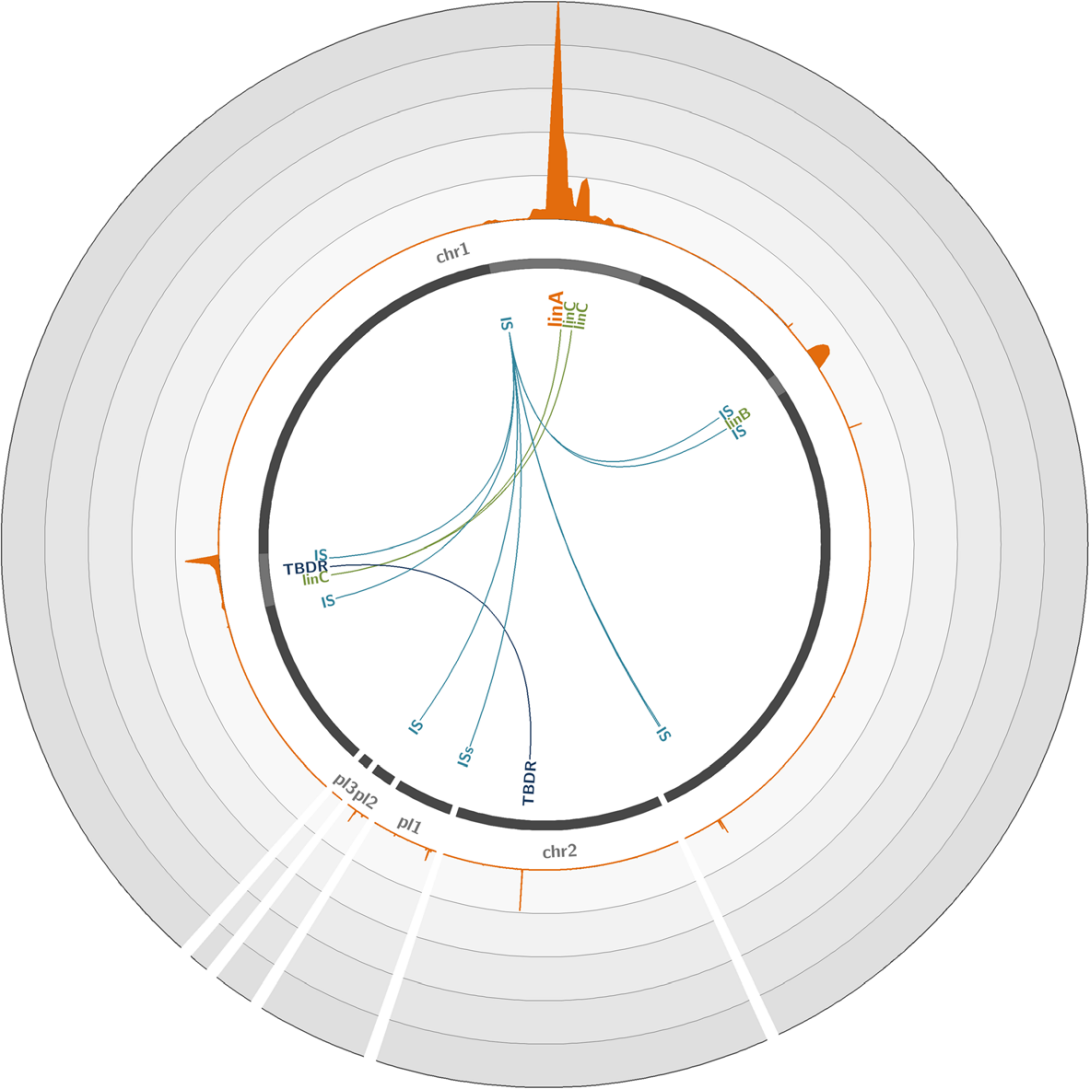
Coverage of the *Sphingobium japonicum* genome with reads obtained through hybridization capture on the artificial mixture of bacterial strains. The *S. japonicum* reference genome (accession number GCA_000091125.1) [37], composed of two chromosomes (chr1 and chr2) and three plasmids (pl 1 to 3), is represented by *dark gray lines*. The *light gray highlights* in the chromosomes represent 45× zoomed regions. The *orange plot* represents the mean number of reads mapped on the genome over a 200-bp window. Each *circle* represents a coverage of 3000 reads per position; the maximum coverage of the genome is 14,956 reads. The positions of significantly enriched genes of interest are indicated, with links representing co-capture events. *IS* IS6100, *TBDR* TonB-dependent receptor

Reference genome mapping analysis of reads revealed an approximate 1400-fold enrichment of *linA*, with 6.72% of sequences being on target (Fig. 1). Due to the capture of long DNA fragments carrying *linA*, we also significantly enriched *linA* flanking regions, totaling 19.15% of sequences. This significant enrichment allowed for the reconstruction of a 21.6-kbp contig spanning *linA* and carrying 19 other genes, including a TonB-dependent receptor, a TetR family transcriptional regulator, an acyltransferase, a flavoprotein, an acyl-CoA dehydrogenase, an insertion sequence 6100 (IS6100), and two copies of the *linC* gene. *linC* encodes a 2,5-dichloro-2,5-cyclohexadiene-1,4-diol dehydrogenase involved after LinA activity in the downstream steps of the HCH degradation pathway (Additional file 1: Figure S2). Our hybridization capture approach also enabled the enrichment of sequences very distant from *linA* in the genome (Fig. 1). Genetic analysis of these regions demonstrated that a co-capture occurred, explaining the enrichment. Through the targeted capture of large DNA fragments, our method enabled the enrichment of *linA* and its adjacent genes, which in turn acted as probes for the enrichment of their homologs located distantly in the *S. indicum* genome. Thereby, with the co-capture of *linC*, we were able to reconstruct an 11-kbp contig containing two copies of *linC* and 8 other flanking genes, including a TonB-dependent receptor, a transposase, three hypothetical proteins, and two IS6100. The co-capture of these mobile elements enabled the enrichment of many other contigs, particularly contigs containing other *lin* genes frequently associated with insertion sequence (IS), such as *linB* and *linD*, which are also involved in HCH degradation (Additional file 1: Figure S2). Thus, we estimated that the captured region containing *linA* and its flanking genes and the co-captured regions total 87.44% of reads, which represents almost all of the sequences obtained after capture and demonstrates the efficiency of the method on a simple model. We were consequently able to efficiently reconstruct more than 40 kbp of the genome of *S. indicum* by targeting the short 471-bp *linA* gene using only four probes.

### Application of hybridization capture to a metagenomic sample

**Fig. 2:**
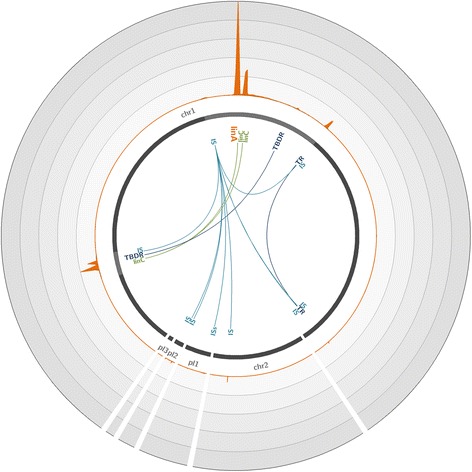
Coverage of the *Sphingobium japonicum* genome with reads obtained through hybridization capture on the metagenomic soil sample. The *S. japonicum* reference genome (accession number GCA_000091125.1) [37], composed of two chromosomes (chr1 and chr2) and three plasmids (pl1 to 3), is represented by *dark gray lines*. The *light gray highlights* in chromosomes represent 45× zoomed regions. The *orange plot* represents the mean number of reads mapped on the genome over a 200-bp window. Each *circle* represents a coverage of 18,000 reads per position; the maximum coverage of the genome is 88,555 reads. The positions of significantly enriched genes of interest are indicated, with links representing co-capture events. *IS* IS6100, *TBDR* TonB-dependent receptor, *TR* transcriptional regulator

**Fig. 3:**
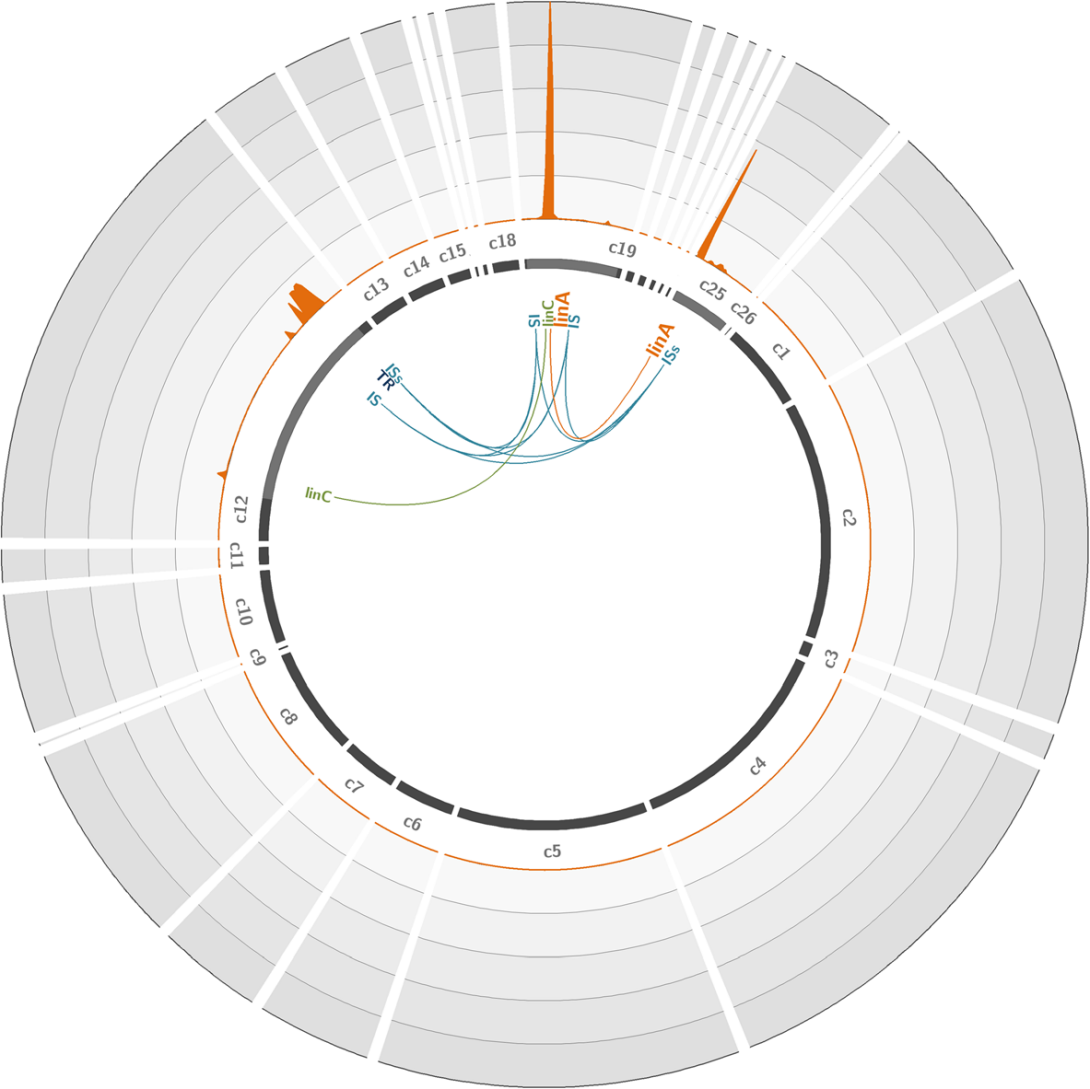
Coverage of the *Novosphingobium barchaimii* genome with reads obtained through hybridization capture on the metagenomic soil sample. The *N. barchaimii* reference draft genome (accession number GCA_001046635.1) [33], composed of 26 contigs (c1 to c26), is represented by *dark gray lines*. The *light gray highlights* in contigs represent 45× zoomed regions. The *orange plot* represents the mean number of reads mapped on the genome over a 200-bp window. Each *circle* represents a coverage of 150,000 reads per position; the maximum coverage of the genome is 732,040 reads. The positions of significantly enriched genes of interest are indicated, with links representing co-capture events. *IS* IS6100, *TR* transcription regulator

We next determined whether our hybridization capture method could scale to metagenomic samples. We used our set of *linA*-targeting probes to hybridize 20-kbp sheared DNA fragments extracted from an HCH-contaminated soil from an old HCH factory to reconstruct large fragments of genomes containing *linA* and to identify microorganisms involved in HCH degradation. We first conducted a de novo assembly of reads obtained from captured products and then affiliated the longest, most covered, and *linA*-carrying contigs. Among enriched sequences, we identified the following seven microbial species of interest carrying the three *linA* types on which we mapped our reads: *S. indicum*, *Sphingobium japonicum*, *Sphingobium baderi*, *Sphingobium* sp. TKS, *Sphingobium* sp. MI1205, *Novosphingobium barchaimii*, and *Sphingomonas* sp. MM-1. Analysis of the coverage of their reference genomes with reads revealed significant *linA* enrichments for all of them compared to shotgun sequencing (Additional file 1: Figure S3 and S4; highly fragmented genomes are not presented). For *S. japonicum*, we obtained a similar pattern to that observed for the mixture of bacterial strains—a significant enrichment in the *linA* gene, its flanking regions containing *linC*, and a co-capture of *linA* surrounding genes at other locations on the genome (Fig. 2). For this species, our approach enabled the reconstruction of a 26.2-kbp contig surrounding *linA* and more than 40 kbp elsewhere in the genome. With this approach, we also reconstructed very large genomic regions surrounding *linA* in *S. indicum* and *N. barchaimii*. For *N. barchaimii*, the two copies of the *linA* genes were enriched simultaneously with *linC*, which is present in its flanking regions, and allowed for the reconstruction of more than 70.0 kbp of the genome of this species (Fig. 3).

**Fig. 4:**
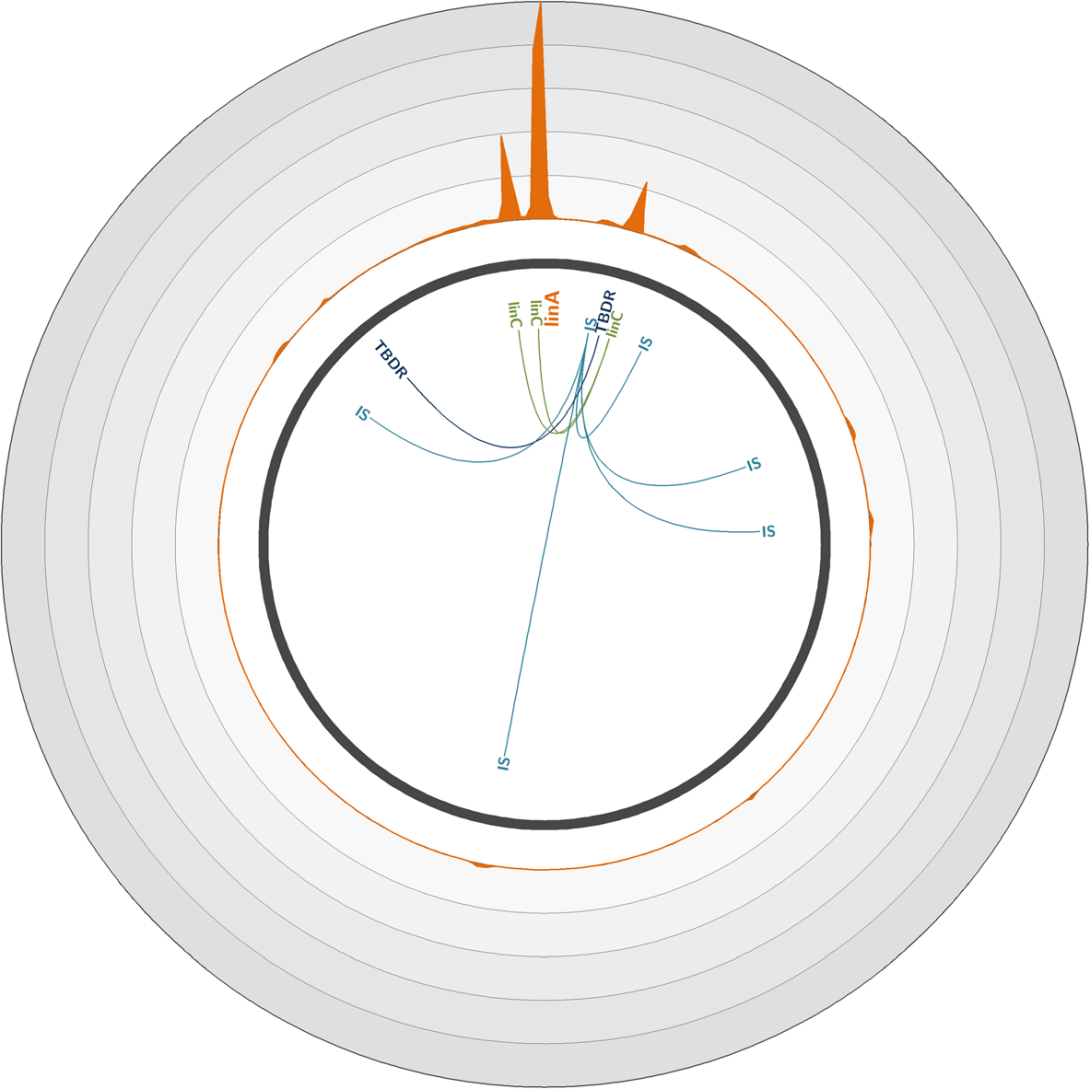
Coverage of the *Sphingobium* sp. TKS plasmid pTK4 with reads obtained through hybridization capture on the metagenomic soil sample. Plasmid pTK4 (accession number CP005088.1) [38] is represented by the *dark gray line*. The *orange plot* represents the mean number of reads mapped on the plasmid over a 200-bp window. Each *circle* represents a coverage of 15,500 reads per position; the maximum coverage of the genome is 76,118 reads. The positions of significantly enriched genes of interest are indicated, with links representing co-capture events. *IS* IS6100, *TBDR* TonB-dependent receptor

Finally, we identified and reconstructed several plasmids carrying *linA*, including pTK4 of *Sphingobium* sp. TKS, pMI2 of *Sphingobium* sp. MI1205, and plSP1 of *Sphingomonas* sp. MM-1. For each plasmid, we significantly enriched *linA* and its flanking regions and reconstructed several tens of kilobase pair contigs. As an example, we completely reconstructed the 75.94-kbp pTK4 plasmid with high coverage (Fig. 4, Additional file 1: Figure S5). Moreover, through co-capture, we were able to identify and reconstruct long fragments of genomes belonging to microorganisms that do not carry the *linA* gene but which contribute to the HCH degradation pathway in downstream steps. Indeed, our approach enabled the assembly of large chromosomal or plasmidic contigs containing *linC* from organisms such as *Sphingomonas* sp. BHC-A, plasmid plSP4 of *Sphingomonas* sp. MM-1, and plasmids pMI1 and pMI3 of *Sphingobium* sp. MI1205. These reconstructed contigs also carry other *lin* genes, such as *linB*, *linD*, *linE*, and *linF*, enabling the identification of species that participate in the HCH pathway degradation after *linA* is utilized (Additional file 1: Figure S2).

## Discussion

Gene capture approaches by hybridization traditionally use tiling probes for resequencing experiments to identify new variants [11]. However, more recent studies have devoted attention to reads outside the targeted regions [28]. Based on the potency of the sequence capture for expanding knowledge beyond target DNA regions, studies have used hybridization capture strategies to study the unknown flanking regions of genes or particular genomic regions [29, 30]. Flanking DNA regions recovered are of small size (around 200 bp) due to the sequencing libraries used for the hybridization step during the capture. Such libraries consist of short lengths of DNA specifically selected for optimal performance with next-generation sequencing platform. In a previous work, we described the first adaptation of hybridization capture to a complex metagenomic sample [12]. This approach efficiently enriched complete targets but also recovered off-target sequences.

In the present study, we developed a new hybridization capture method that facilitates the reconstruction of large genomic regions surrounding a targeted biomarker from both a simple biological model and complex metagenomes. Owing to the significant and specific enrichment in several tens of kilobase pairs allowed using this approach, we were able to reconstruct large DNA regions by only targeting a few tens of base pairs with the use of a reduced set of specific non-overlapping probes. This targeted sequencing for the reconstruction of large unknown genomic regions is either impossible or requires huge effort and cost using other conventional molecular approaches, such as direct sequencing. With our strategy, it is possible to easily access unexplored or difficult-to-explore genomic regions of single organisms or organism communities. Considering metagenomic samples, parts or complete genomes of targeted microorganisms, even from the rare biosphere, could be revealed. Through this hybridization capture approach, we were able to rapidly reconstruct many contigs longer than 20 kbp carrying the targeted marker from a soil sample considered to be the most complex ecosystem in terms of microbial diversity. Thus, we were able to assemble large portions of chromosomes and even complete plasmids harboring the target. Through co-capture, we were also able to identify species not harboring the targeted marker but potentially involved in the biodegradation process because of the presence of genes encoding enzymes acting downstream of LinA dehydrochlorination. Comparative metagenomic analyses revealed complex microbial structure of HCH-contaminated soils, but precise description of microbial populations harboring *lin* genes remains too difficult in such complex ecosystem [31]. Other studies focused on specific microbial populations to describe biodegradation metabolic capacities by genomics approaches [32–34]. Therefore, it is possible to use our hybridization capture approach to associate the identities of the microorganisms present and the specific metabolic functions they perform with certainty, which is the ultimate goal in microbial ecology. Applied to our study model, this method enabled an unambiguous identification of several bacterial species harboring metabolic capacities for HCH degradation, which constitutes valuable information for conducting efficient soil bioremediation by biostimulation approaches [35].

In this way, we have been able to identify seven bacterial species carrying *linA* that had already been described for their HCH degradation capabilities. However, applied to this contaminated soil sample, this new hybridization capture strategy has not evidenced the presence of previously unknown HCH-degrading organisms through the enrichment of *linA*. Given the demonstrated efficiency of this new method and its sensitivity, we can easily imagine the identification of new species involved in HCH degradation in other soils contaminated with the pollutant. Indeed, because of their frequent presence on plasmids and association with IS6100, as is the case in this study, *lin* genes are often submitted to horizontal gene transfer (HGT) promoting their acquisition by other microorganisms [34, 36]. Our method could consequently be a relevant tool to study the mobility of *lin* genes in HCH-contaminated sites. For instance, through the enrichment of IS6100, hybridization capture could improve the comprehension of the transfer of all the IS6100-associated *lin* genes among microbial communities or within a particular genome through homologous recombination without requiring the isolation of microorganisms of interest [33]. Similarly, by applying this method to many contaminated soils, this hybridization capture method could help to go further in evolution studies by revealing new *lin* gene variants never described before.

Finally, using our approach, the targeted reconstruction of complete genomes from metagenomic samples becomes possible and microbial dark matter genomes could be revealed. Thus, with few rounds of capture cycles using probes designed on the reconstructed regions to expand or by directly targeting nucleic sequences scattered throughout the genome of interest, the reconstruction of complete genomes from metagenomic samples can be rapidly achieved. This strategy can also be adapted for the finishing step of complete genome reconstruction from draft reference genomes. We used sequencing data obtained from the mixture of strains and the soil sample to demonstrate that joining existing contigs from draft genomes is possible owing to the enriched large regions that span the targeted biomarker. For example, we were able to associate small contigs harboring *linA* with other contigs for reference genomes from *S. indicum* and *N. barchaimii*. Direct genome sequencing on isolated bacteria could not resolve this genomic region due to the presence of ISs close to the targeted marker. Moreover, coupling our hybridization capture method with the use of third-generation sequencing platforms, such as PacBio sequencing, would enable the direct sequencing of the long fragments captured and would consequently further improve the resolution of complex DNA regions harboring numerous repeats.

## Conclusions

Despite the advent of NGS technologies, de novo assembly of whole genomes from single organisms or metagenomic samples remains a huge challenge, with numerous genomic regions of interest that still remain underexplored. Here, we present a new hybridization capture strategy that allows the enrichment of large genomic regions of tens of kilobase pairs, through targeting a short DNA sequence. Applied to a metagenomic soil sample, this method allowed to reconstruct many contigs longer than 20 kbp carrying the 471-bp targeted functional biomarker. Thus, we identified species potentially involved in the metabolic process of interest. This innovative strategy opens new possibilities to expand knowledge beyond known targeted DNA regions for both organism communities and single organisms and resolve concerns in numerous biological studies.

## Additional files

Additional file 1: Word document that includes supplemental Figures S1 to S5 and Table S1. Table S1. Set of probes used for hybridization capture targeting *linA*. Figure S1. Schematic representation of large DNA fragment hybridization capture method. Figure S2. Hexachlorocyclohexane (HCH) degradation pathways. Figure S3. Coverage of the *Sphingobium japonicum* genome with reads obtained through hybridization capture and shotgun sequencing on the metagenomic soil sample. Figure S4. Coverage of the *Novosphingobium barchaimii* genome with reads obtained through hybridization capture and shotgun sequencing on the metagenomic soil sample. Figure S5. Coverage of the *Sphingobium* sp. TKS plasmid pTK4 with reads obtained through hybridization capture and shotgun sequencing on the metagenomic soil sample. (DOCX 1092 kb)

## Abbreviations

HCH: Hexachlorocyclohexane
IS: Insertion sequence
MDA: Multiple displacement amplification
NGS: Next-generation sequencing
SRA: Sequence read archive
