# Supplementary material for: Revealing large metagenomic regions through long DNA fragment hybridization capture
Source: Microbiome. 2017 Mar 14;5:33. doi: 10.1186/s40168-017-0251-0 (PMC5351058; doi:10.1186/s40168-017-0251-0)
Supplement: Additional file 1: — Word document that includes supplemental Figures S1 to S5 and Table S1. Table S1. Set of probes used for hybridization capture targeting linA. Figure S1. Schematic representation of large DNA fragment hybridization capture method. Figure S2. Hexachlorocyclohexane (HCH) degradation pathways. Figure S3. Coverage of the Sphingobium japonicum genome with reads obtained through hybridization capture and shotgun sequencing on the metagenomic soil sample. Figure S4. Coverage of the Novosphingobium barchaimii genome with reads obtained through hybridization capture and shotgun sequencing on the metagenomic soil sample. Figure S5. Coverage of the Sphingobium sp. TKS plasmid pTK4 with reads obtained through hybridization capture and shotgun sequencing on the metagenomic soil sample. (DOCX 1092 kb) [file 40168_2017_251_MOESM1_ESM.docx]

**Additional file**

**Revealing large metagenomic regions through long DNA fragment hybridization capture**

Cyrielle Gasc^1^ and Pierre Peyret^2,^*

^1^ Université Clermont Auvergne, INRA, MEDIS, F-63000 Clermont Ferrand, France

* Correspondence: pierre.peyret@uca.fr

**Supplementary table and figures:**

**Table S1.** Set of probes used for hybridization capture targeting *linA.*

**Figure S1.** Schematic representation of large DNA fragment hybridization capture method.

**Figure S2.** Hexachlorocyclohexane (HCH) degradation pathways.

**Figure S3.** Coverage of the *Sphingobium japonicum* genome with reads obtained through hybridization capture and shotgun sequencing on the metagenomic soil sample.

**Figure S4.** Coverage of the *Novosphingobium barchaimii* genome with reads obtained through hybridization capture and shotgun sequencing on the metagenomic soil sample.

**Figure S5.** Coverage of the *Sphingobium* sp. TKS plasmid pTK4 with reads obtained through hybridization capture and shotgun sequencing on the metagenomic soil sample.

**Table S1**. Set of probes used for hybridization capture targeting *linA*. Bold characters in probe sequences represent amplification adapters. Probe lengths refer to the size of the *linA*-specific sequence.

| **Probe**  **name** | **Sequence** | **Position**  **(bp)** | **Size**  **(bases)** | **Degeneracy** |
| --- | --- | --- | --- | --- |
| **linA_1** | **ATCGCACCAGCGTGT**ATGAGTGATCTAGACAGACTYGCAAGCCGGGCYGCGATTCAGGACCTCTACTCTGACMAGCTCATTGSCGTAGWCAAGCG**CACTGCGGCTCCTCA** | 1-80 | 80 | 32 |
| **linA_2** | **ATCGCACCAGCGTGT**AGAGTGGACCRTTGAGGGAATCGGCMCCTACAAGRGYCCGGAAGGCGCSCTCGATTTGGYCAATAACGTANTCTGGCCAA**CACTGCGGCTCCTCA** | 120-199 | 80 | 256 |
| **linA_3** | **ATCGCACCAGCGTGT**TGGAATTTGTGAGCGCGGACAWGGTAAATGGTATTGGCGACGTCCTTYKCCTYGGAAATCTCGTCGAAGSTAATCAGTCG**CACTGCGGCTCCTCA** | 239-319 | 80 | 32 |
| **linA_4** | **ATCGCACCAGCGTGT**GCGCCGTGACGGGGTGTGGAAGYTCTYTAAGCBCAACGBATGCAYGAACTATTTCACCCCGCWGGCCGGYATTCATTTCG**CACTGCGGCTCCTCA** | 354-433 | 80 | 288 |

**
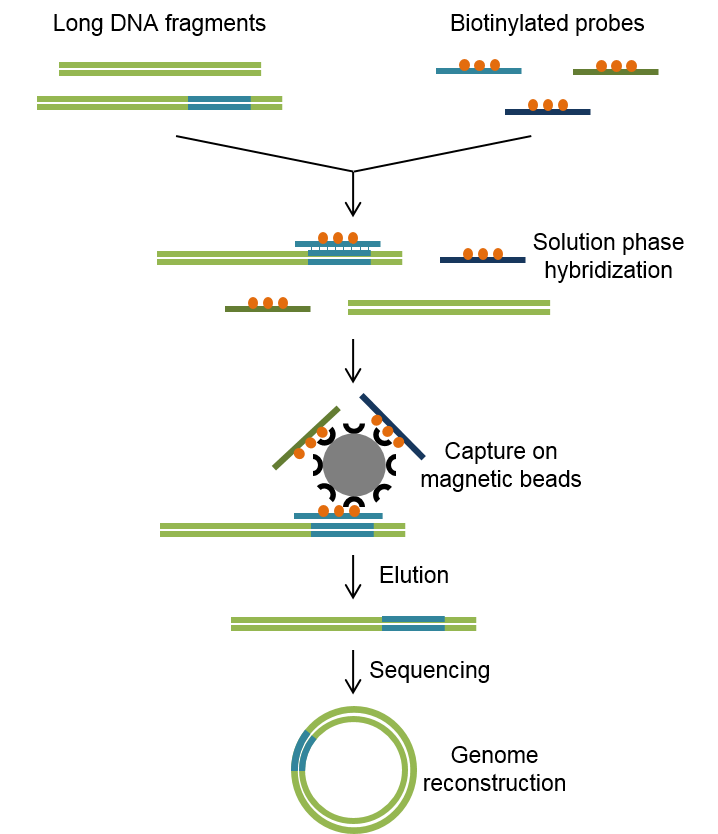
**

**Figure S1**. Schematic representation of large DNA fragment hybridization capture method. Several-kbp DNA fragments containing a targeted biomarker are hybridized against a set of short specific biotinylated probes. Hybridization is performed in solution, and long DNA fragment-probe heteroduplexes are captured using streptavidin-coated magnetic beads through interaction with biotin. Non-target sequences are washed away, and the enriched sample is eluted and sequenced.

**
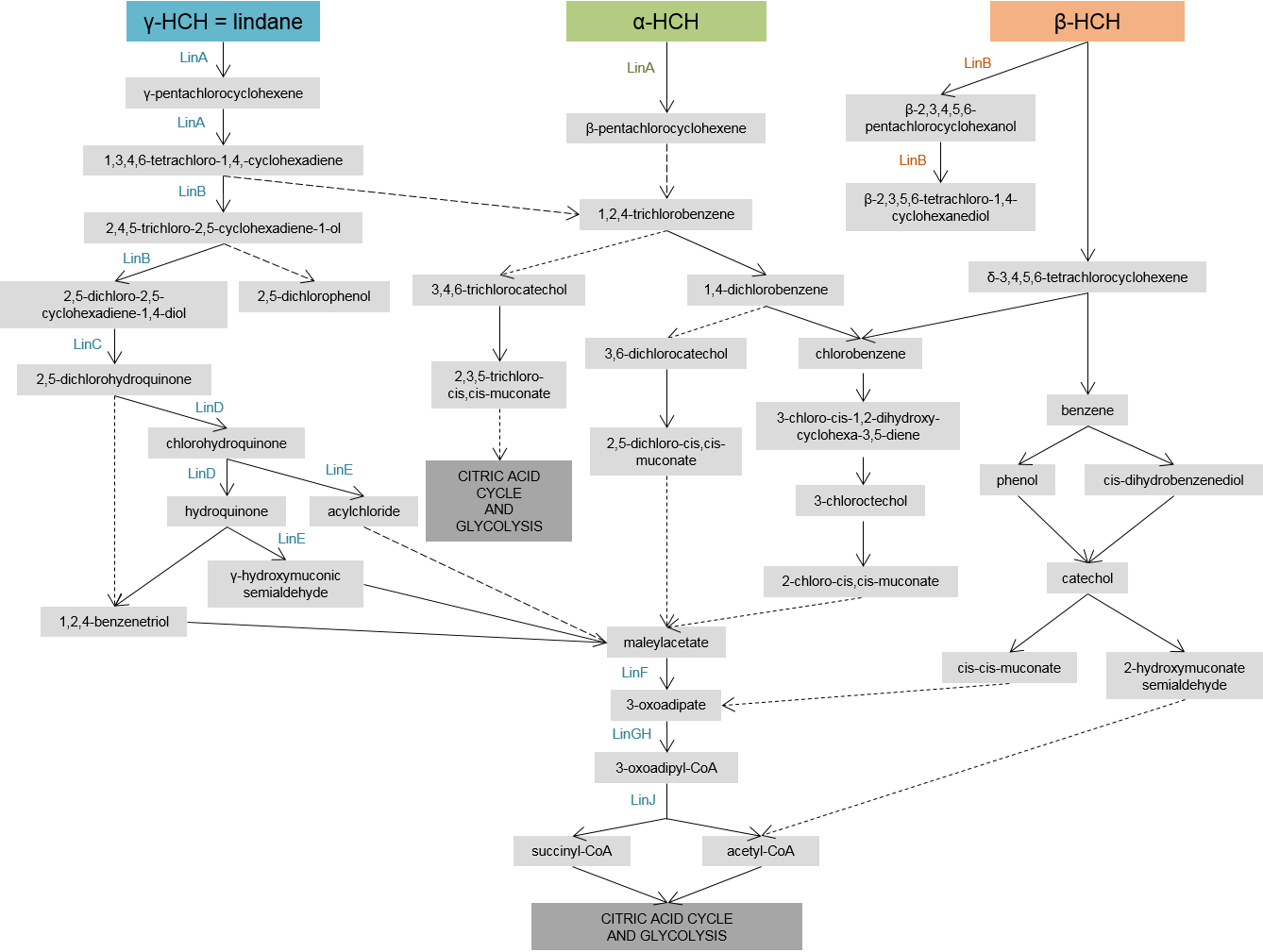
**

**Figure S2**. Hexachlorocyclohexane (HCH) degradation pathways. HCH is composed of a mixture of different isomers, including α-, β- and γ-HCH. Its degradation involves *linA* to *linJ* gene products.

**
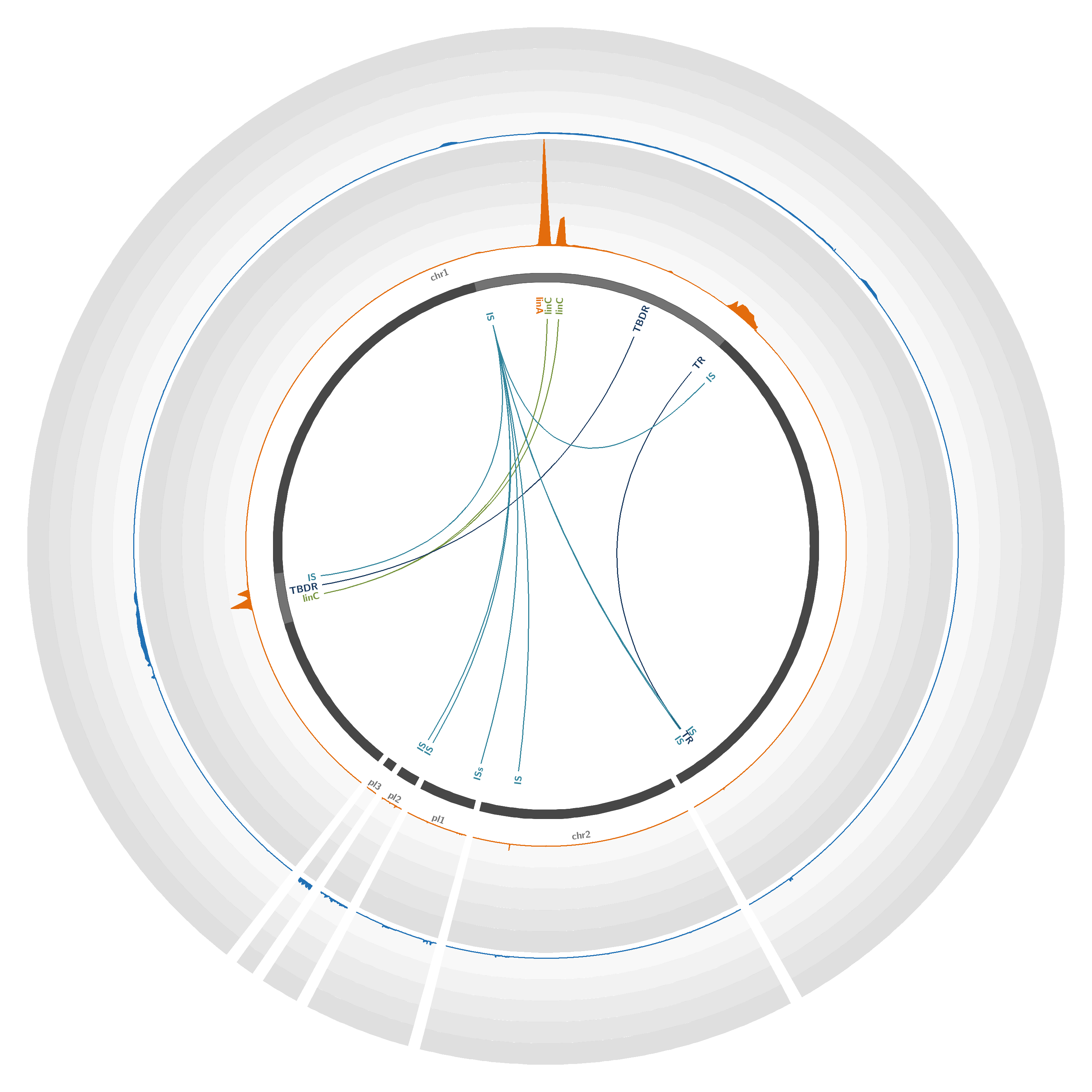
**

**Figure S3**. Coverage of the *Sphingobium japonicum* genome with reads obtained through hybridization capture and shotgun sequencing on the metagenomic soil sample. The *S. japonicum* reference genome (accession number GCA_000091125.1) [1], composed of two chromosomes (chr1 and chr2) and three plasmids (pl 1 to 3), is represented by dark gray lines. The light gray highlights in chromosomes represent 45X-zoomed regions. The orange and blue plots represent the mean number of reads obtained through hybridization capture and shotgun sequencing, respectively, mapped on the genome over a 200-bp window. Each circle represents a coverage of 18,000 reads per position; the maximum coverage of the genome is 88,555 reads. The positions of significantly enriched genes of interest are indicated, with links representing co-capture events. IS = IS6100, TBDR = TonB-dependent receptor, TR = Transcriptional regulator.

**
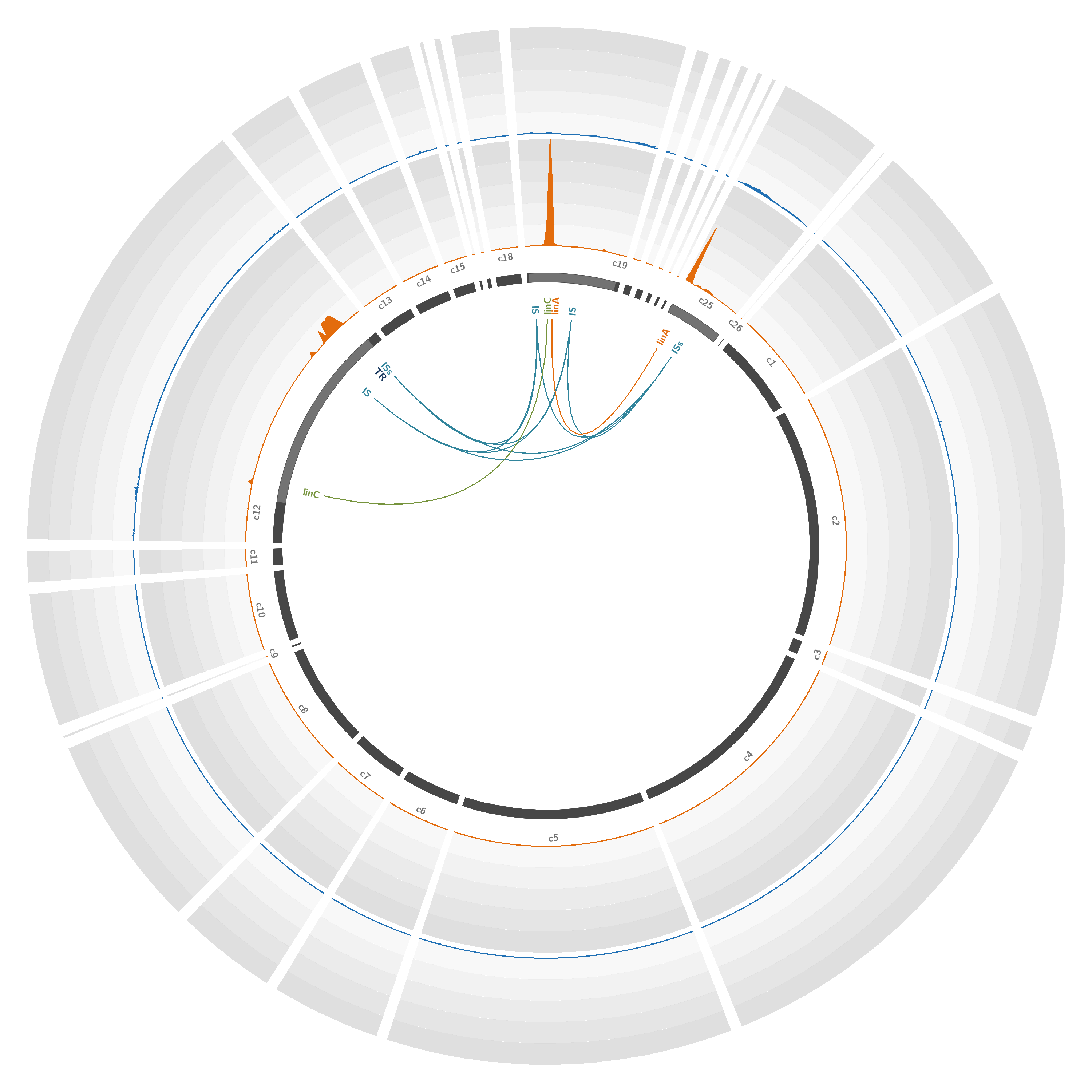
**

**Figure S4**. Coverage of the *Novosphingobium barchaimii* genome with reads obtained through hybridization capture and shotgun sequencing on the metagenomic soil sample. The *N. barchaimii* reference genome (accession number GCA_001046635.1) [2], composed of 26 contigs (c1 to c26), is represented by dark gray lines. The light gray highlights in contigs represent 45X-zoomed regions. The orange and blue plots represent the mean numbers of reads obtained through hybridization capture and shotgun sequencing, respectively, mapped on the genome over a 200-bp window. Each circle represents a coverage of 150,000 reads per position; the maximum coverage of the genome is 732,040 reads. The positions of significantly enriched genes of interest are indicated, with links representing co-capture events. IS = IS6100, TR = transcription regulator.

**
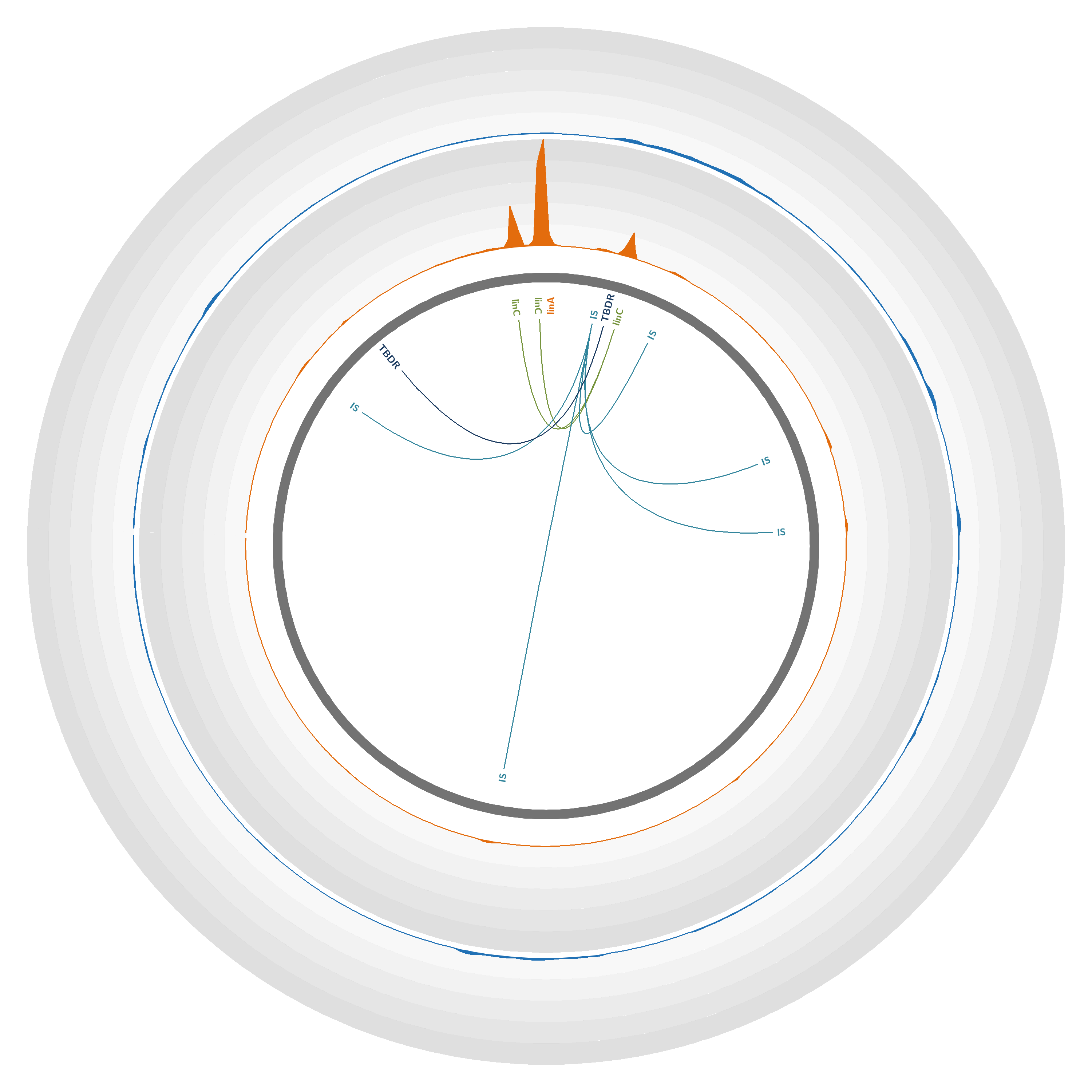
Figure S5**. Coverage of the *Sphingobium sp.* TKS plasmid pTK4 with reads obtained through hybridization capture and shotgun sequencing on the metagenomic soil sample. Plasmid pTK4 (accession number CP005088.1) [3] is represented by the dark gray line. The orange and blue plots represent the mean numbers of reads obtained through hybridization capture and shotgun sequencing, respectively, mapped on the genome over a 200-bp window. Each circle represents a coverage of 15,500 reads per position; the maximum coverage of the plasmid is 76,118 reads. The positions of significantly enriched genes of interest are indicated, with links representing co-capture events. IS = IS6100, TBDR = TonB-dependent receptor.

**References**

1. Nagata Y, Ohtsubo Y, Endo R, Ichikawa N, Ankai A, Oguchi A, Fukui S, Fujita N, Tsuda M: Complete genome sequence of the representative gamma-hexachlorocyclohexane-degrading bacterium Sphingobium japonicum UT26. *J Bacteriol* 2010, 192:5852-5853.

2. Pearce SL, Oakeshott JG, Pandey G: Insights into Ongoing Evolution of the Hexachlorocyclohexane Catabolic Pathway from Comparative Genomics of Ten Sphingomonadaceae Strains. *G3 (Bethesda)* 2015, 5:1081-1094.

3. Tabata M, Ohhata S, Kawasumi T, Nikawadori Y, Kishida K, Sato T, Ohtsubo Y, Tsuda M, Nagata Y: Complete Genome Sequence of a Gamma-Hexachlorocychlohexane Degrader, Sphingobium sp. Strain TKS, Isolated from a Gamma-Hexachlorocychlohexane Degrading Microbial Community. *Genome Announcements* 2016, 4:e00247-00216.
